# Clinical, behavioural and pharmacogenomic factors influencing the response to levothyroxine therapy in patients with primary hypothyroidism—protocol for a systematic review

**DOI:** 10.1186/s13643-017-0457-z

**Published:** 2017-03-21

**Authors:** Rosie Dew, Onyebuchi Okosieme, Colin Dayan, Vinay Eligar, Ishrat Khan, Salman Razvi, Simon Pearce, Scott Wilkes

**Affiliations:** 1 University of Sunderland, City Campus, Chester Road, Sunderland, SR1 3SD UK; 2 Prince Charles Hospital, Cwm Taf University Health Board, Merthyr Tydfil, CF47 9DT UK; 3 Cardiff University School of Medicine, Sir Geraint Evans Building, Heath Park Cardiff, CF14 4XN UK; 4 International Centre for Life, Newcastle University, Central Parkway, Newcastle upon Tyne, NE1 3BZ UK

**Keywords:** Primary hypothyroidism, Subclinical hypothyroidism, Levothyroxine, TSH

## Abstract

**Background:**

Suboptimal thyroid hormone therapy including under-replacement and over-replacement is common amongst patients with hypothyroidism. This is a significant health concern as affected patients are at risk of adverse cardiovascular or metabolic consequences. Despite a growing body of evidence on the effects of various factors on thyroid hormone replacement, a systematic appraisal of the evidence is lacking. This review aims to appraise and quantify the extent to which clinical, behavioural and pharmacogenomic factors affect levothyroxine therapy in patients with primary hypothyroidism.

**Methods/design:**

The databases Web of Science, Cochrane Library, EMBASE and PubMed will be searched. Patients must be adults over the age of 18 years, suffering from primary hypothyroidism including overt and subclinical hypothyroidism and receiving levothyroxine treatment. Studies in children, pregnant women and patients with secondary or tertiary hypothyroidism will not be included. We will also exclude studies focused on forms of thyroid hormone replacement therapy other than levothyroxine.

The primary outcome is to quantify the effect of clinical, behavioural and pharmacogenomic factors on thyroid stimulating hormone (TSH) levels. Secondary outcomes are the effect these factors have on thyroxine (T4) and triiodothyronine (T3) levels, mortality, morbidity, quality of life, treatment complications, adverse effects, physical and social functioning. Studies will be screened through reading the title, abstract and then full text. Two reviewers will independently extract the data and select articles, and a third reviewer will be consulted if there is any disagreement. We will undertake a meta-analysis of studies in which there is a defined intervention or exposure, patients are receiving levothyroxine for hypothyroidism, there is an appropriate control group of levothyroxine treated patients that are not exposed to the intervention, and the primary outcome is determined by serum TSH levels. Studies will comprise of randomised controlled trials as well as observational data.

Eligible studies will be assessed for bias using the risk of bias tool available in the Cochrane handbook 2011, and the quality of evidence will be judged according to the Grading of Recommendations Assessment, Development, and Evaluation (GRADE) approach. A flow diagram describing the data search will be created according to the Preferred Reporting Items for Systematic Reviews and Meta-Analysis: The PRISMA statement. A narrative synthesis will be undertaken in the description of the data, and summary tables will be created of the results.

**Discussion:**

This review will be the first systematic review of this nature. The evidence synthesised will be useful to general practitioners in their management of hypothyroidism. Findings will be disseminated at conferences and in professional and peer-reviewed journals.

**Systematic review registration:**

PROSPERO CRD42015027211

**Electronic supplementary material:**

The online version of this article (doi:10.1186/s13643-017-0457-z) contains supplementary material, which is available to authorized users.

## Background

Hypothyroidism is a common disease affecting 3–5% of the population and is the result of insufficient production of thyroid hormones. Over 99% of hypothyroid cases are caused by primary hypothyroidism or inadequate function of the thyroid gland [1, 2]. Within the thyroid disease free population, the reference range for thyroid stimulating hormone, thyrotropin (TSH), is commonly between 0.4 and 4.0 mU/L, and levels above this are indicative of hypothyroidism. Overt hypothyroidism is a clinical condition in which TSH is increased above the reference range, and free thyroid hormones, most significantly thyroxine (T4), are low, while subclinical hypothyroidism (SCH) refers to TSH levels above the normal reference range, but free thyroid hormones are within their reference range [3].

Three percent of the UK population receive thyroid hormone replacement therapy [4]. Synthetic levothyroxine is commonly used, and the goal of therapy is to achieve clinical wellbeing and restore serum TSH levels to within the reference range. Levothyroxine has a long half-life of about 7 days, and so, a once daily dose provides stable and relatively constant serum hormone levels. With individual dosage adjustment, levothyroxine replacement therapy is safe and well tolerated [5]. However, over a third of patients with hypothyroidism are inadequately treated i.e. under-treated or over-treated, as shown by abnormal serum TSH levels in community-based cohorts of levothyroxine treated patients [6, 7]. This problem has persisted for decades and remains an issue even with frequent biochemical monitoring of patients [8–10]

Patients with inadequate replacement have an increased risk of cardiovascular events, fractures [11–13], dyslipidaemia [14], neurocognitive dysfunction [15] and in extreme cases, may develop the life threatening state of myxoedema coma [16]. In addition, there are healthcare resource implications of having abnormal thyroid biochemistry as these patients are more likely to need their blood tests repeated, have frequent adjustments to their levothyroxine dose, experience recurrent symptoms affecting well-being and quality of life, and contribute to prescription wastage from poor adherence to treatment. Reduced quality of life is very common amongst hypothyroid patients, particularly relating to energy, motivation, physical capabilities, physical appearance and weight [17]. Furthermore, hypothyroid patients even with apparently normal TSH levels report having reduced psychological well-being [18] and poor quality of life [19]. To what extent thyroid hormone levels have a causative role in the symptoms in these cases remains to be determined.

There is now a growing body of evidence describing the effects of a variety of factors on thyroid hormone therapy. Some of these factors include body weight, pregnancy, co-morbid conditions, consistency and quality of levothyroxine, drug interactions and dose timings, and behavioural factors such as adherence rates. Pharmacokinetic factors also play a role since levothyroxine is absorbed from the stomach, and small bowel and its optimal absorption is dependent on the acidic environment of the stomach. Several factors are known to perturb absorption through this mechanism, including the use of calcium or iron salts, proton pump inhibitors, atrophic gastritis (pernicious anaemia) and coeliac disease [20, 21]. Pharmacogenomic associations may also be relevant to the adequacy of thyroxine therapy, and the evidence in this area is increasing. For example, within the metabolic pathway of thyroxine, polymorphisms in the type 2 deiodinase, DIO2, (Thr92Ala) has been shown to influence the levothyroxine dose required to achieve target TSH levels [22].

Thus, there will undoubtedly be multifactorial reasons for poor response to therapy in patients with hypothyroidism. This review aims to summarise the contemporary literature and quantify the extent that clinical, behavioural and pharmacogenomic factors affect the response to levothyroxine and contribute to abnormal TSH levels in patients with primary hypothyroidism.

## Methods/design

The systematic review including its methodology will be reported according to the Preferred Reporting Items for Systematic Reviews and Meta-Analysis: The PRISMA statement [23]. The PRISMA statement refers to a checklist, which includes items deemed essential for precise reporting of a systematic review. The additional file shows this in more detail (Additional file 1).

Since there are a variety of factors associated with the response to levothyroxine therapy, this review will adopt a broad approach to answering the review question. Eligibility criteria will ensure hypothyroid patients as the population of interest and the effect on TSH as the primary outcome focus. A summary of participants, interventions, comparators, outcomes and study design (PICOS) details are outlined in Table 1.

**Table 1:** Summary of the PICOS elements included in the systematic review

	Inclusion criteria	Exclusion criteria
Population/patients	Human subjects	Animals
	18 years or older	Children
	Patients with diagnosed primary hypothyroidism or subclinical hypothyroidism	Pregnant women/patients with secondary or tertiary hypothyroidism/hyperthyroid patients/patients previously treated for thyroid cancer
	Receiving levothyroxine treatment	Other forms of hormone replacement therapy/combination therapy
Intervention	Interventions of any type, intensity and frequency, with length greater than 6 weeks used to investigate effect of clinical, behavioural or pharmcogenomic factors on levothyroxine therapy	N/A
Comparators/control	A control or comparative group is necessary. This can be placebo/no treatment/standard therapy/usual care/alternative treatment	No control/comparative group
Outcome	Primary outcome; quantitative effect of clinical/behavioural/pharmacogenomic factors on TSH levels Secondary outcome (s); quantitative effect of clinical/behavioural/ Pharmacogenomics factors on T4 and T3 levels/any short term effects/mortality/morbidity/quality of life/treatment complications/adverse effects/rate of relapse/physical functioning/social functioning	Reviews only focused on the quantitative effect of clinical/behavioural/ Pharmacogenomics factors on T4 and T3 levels
Study design	RCTs, case-control studies, cohort studies, observational studies, cross sectional studies, longitudinal studies, case studies	N/A

### Types of studies

Findings from preliminary searches using the keywords hypothyroidism, TSH, food, comorbid, concomitant, compliance, levothyroxine, drugs and OATP1, MCT, UGT, FOXE and deiodinase genes in Web of Science database showed a large variation in the study design used in articles, and the articles consist mainly of non-randomised studies (NRT). Following Cochrane handbook guidance, risk of bias, confounding and heterogeneity will be considered for the type of studies to be used in our review [24]. Since our systematic review needs to have a broad approach to cover a wide range of factors, all studies will be included within our search; RCTs, case-control, cohort, cross sectional, longitudinal, observational and case studies. This will enable evaluation of interventions that were not studied using a RCT design and therefore allows a wide spread of data to be analysed. However, we will take into account design weaknesses as well as the potential for selection bias, outcome reporting bias and confounding of which all NRS are at risk. We will include full journal articles published in English with sufficient data presented in abstracts. Studies will be required to compare TSH levels of hypothyroid patients with or without the interventions defined below.

### Participants

Human participants aged 18 years or older diagnosed with primary hypothyroidism, whether overt or subclinical, and receiving levothyroxine therapy. Exclusion criteria are cited in Table 1.

### Interventions

Interventions addressing factors that may affect the adequacy of levothyroxine therapy in the hypothyroid population will be included. Selected studies will involve interventions of any type, frequency and intensity that modify clinical, pharmacological or behavioural factors, including studies based on pharmacogenomic characteristics that may affect levothyroxine availability. Such interventions will include measures that influence thyroxine pharmacokinetics, such as drug administration dosage and scheduling, drug interactions, management of co-morbid conditions, and measures designed at improving medication adherence. Interventions will be required to have duration of greater than 6 weeks, and the effectiveness of the intervention on levothyroxine therapy will be measured by TSH levels. Interventions are grouped as:

- Concomitant medications taken with levothyroxine: proton pump inhibitors, omeprazole, lansoprazole, lanthanum carbonate, calcium, antacids, sevelamer hydrochloride, cholestyramine, colsevelam, ferrous sulphate and aluminium hydroxide.
- Behavioural factors that could affect levothyroxine: dose timing, compliance, adherence, attitudes and perceptions.
- Co-morbidities present in hypothyroid patient: lactose intolerance, coeliac disease, gastritis, type 2 diabetes, pancreatic disease, liver disease and pernicious anaemia
- Pharmacogenomic factors that may impact on levothyroxine bioavailability: OATP1, MCT, UGT, FOXE and deiodinase genes

Studies must report results of the effects of interventions on TSH levels, and if also provided T4 and Triiodothyronine (T3) levels, as well as the effects of interventions on other secondary outcomes described in Table 1. Reports of thyroid hormone concentrations will be based on biochemical analysis of participant blood samples using standardised assay methods. PCR will be used for detection of single nucleotide polymorphisms in thyroid genes. Interventions reporting quality of life will be based on standardised questionnaires, including generic (e.g. SF-36) or disease specific questionnaires (e.g. ThySRQ and ThyTSQ).

A *p* value of less than 0.05 will be used to assess whether an intervention has had a significant effect on levothyroxine therapy. This will apply for TSH, T4 and T3 levels as well as quality of life and other secondary outcomes as described in Table 1, if provided in an eligible article. Summary values of outcomes will be reported in our systematic review and include means plus standard deviation and medians plus ranges where provided. Differences in summary values will be discussed in the systematic review to assess the effect of factors on levothyroxine therapy. Comparisons will report factors that have an effect on levothyroxine therapy compared to others.

### Comparator/control

A control or comparator group is required for studies to be included in this review, to ensure no potential bias, experimental errors and to give a baseline or final comparison.

### Search strategy and subject index terms

The keywords hypothyroidism, TSH, food, comorbid, concomitant, compliance, levothyroxine, drugs and OATP1, MCT, UGT, FOXE and deiodinase genes were used in a preliminary search of Web of Science and obtained a total of 41,242 articles. The results of this search were evaluated for relevance, and the search terms were refined accordingly and will be adapted to the respective database. Due to the broad nature of this systematic review, six search strategies have been developed and are displayed in Table 2 using PubMed as an example. Following this, a more in-depth search of databases EMBASE, Web of Science, Cochrane Library and PubMed will be undertaken. In addition to articles found from database searches, relevant articles will also be identified from reference lists of publications.

**Table 2:** Search strategy example used in PubMed database

Number	Search terms	Number of articles
1	Hypothyroidism OR thyroid stimulating hormone OR TSH OR levothyroxine OR LT4 OR thyroid replacement therapy [Title] [Abstract]	5443
2	Food OR Soy OR Coffee OR Caffeine OR Fibre [Title] [Abstract]	65014
3	Comorbid OR comorbidity OR lactose intolerance OR coeliac disease OR celiac disease OR pernicious anaemia OR Vitamin B12 deficiency OR anaemia gastritis OR type 2 diabetes OR pancreatic disease OR pancreatitis OR liver disease [Title] [Abstract]	62790
4	Concomitant OR compliance OR adherence OR levothyroxine AND dose [Title] [Abstract]	56842
5	Attitudes OR perceptions [Title] [Abstract]	26370
6	Co-administration OR proton pump inhibitors OR omeprazole OR lansoprazole OR lanthanum carbonate OR calcium AND medication OR antacids OR sevelamer hydrochloride OR cholestyramine OR colesevelam OR ferrous sulphate OR aluminium hydroxide [Title] [Abstract]	4819
7	OATP1 OR MCT OR UGT OR FOXE OR deiodinase OR polymorphism [Title] [Abstract]	32483
8	1 AND 2	890
9	1 AND 3	205
10	1 AND 4	202
11	1 AND 5	10
12	1 AND 6	18
13	1 AND 7	153

### Outcome measures

#### Primary outcome

The primary outcome of this review is to identify and quantify the effect of the listed interventions (clinical, behavioural and pharmacogenomic) on TSH levels.

#### Secondary outcomes

The secondary outcomes (if documented) will be to quantify the effect of the listed interventions on T4 and T3 levels. Additional secondary outcome measures will include any effects upon mortality, morbidity, quality of life, treatment complications, adverse effects, physical functioning and social functioning.

### Data extraction and synthesis

One review author (RD) will screen titles and abstracts and remove duplicates. Two reviewers (RD and OO) will then screen titles and abstracts against inclusion/exclusion criteria. Studies to be included in the review will be agreed between the two screening reviewers. If there is disagreement between the reviewers, a third reviewer (SW) will be consulted. A Preferred Reporting Items for Systematic Reviews and Meta-Analysis (PRISMA) [23] flow chart will be constructed. We will construct a data extraction form and two reviewers (RD and OO) will extract the data independently and populate the database. A third reviewer (CMD) may be required for consensus.

The following data will be extracted from articles that meet the inclusion criteria:

1. Authors, year of publication, country, study design and number of patients
2. Population demographics
3. Aetiology of hypothyroidism in patient population
4. Co-morbidities in patient population
5. Levothyroxine dose—range/average for each patient group
6. TSH levels—range/average for each patient group
7. Intervention, type, frequency and duration

### Risk of bias (quality) assessment

Two reviewers (RD and OO) will independently assess each eligible study for risk of bias using the risk of bias tool in the Cochrane handbook 2011 [24]. This covers random sequence generation (selection bias), allocation concealment (selection bias), blinding of participants and personnel (performance bias), blinding of outcome assessment (detection bias, patient-reported outcomes bias and mortality bias), incomplete outcome data addressed (attrition bias) and selective reporting (reporting bias). Other forms of bias such as study design bias and response bias will also be assessed by the reviewers. For missing data, authors of the eligible studies will be contacted to see if relevant data can be obtained and used in this systematic review.

### Quality of evidence assessment

Eligible articles will be assessed for quality of evidence referring to the Grading of Recommendations Assessment, Development, and Evaluation (GRADE) guidelines [25]. Initial ranking of the quality of a study based on study design, data collection and data analysis will precede downgrading or upgrading of study quality taking into account limitations and effect sizes according to the Cochrane handbook 2011 [24]. A final grade will then be applied and a score of ‘high’, ‘moderate’, ‘low’ or ‘very low’ will be given to studies as a measure of the quality of evidence. Any disagreement between reviewers will be resolved by consulting with a third reviewer (SW).

### Strategy for data synthesis

The four-phase flow diagram created will depict the search strategy used during the review, and the numbers of articles excluded and included, and on what basis (PRISMA) [23]. A narrative synthesis of the findings from the included studies will be provided during this review. Descriptive summary tables will also be created, including a summary of all the studies included in the review and the design and quality assessments of these studies. The effect measures for the primary and secondary outcomes will be summarised. For the main outcome, effect measures will be the difference in mean or median TSH and also T3 and T4 where provided. Effect measures for quality of life (QoL) outcomes will be the summary difference in QoL questionnaire scores in the intervention and control arms. For other outcomes such as morbidity and mortality rates, effect measures will be the odds ratios (OR) or relative risks (RR) provided.

### Meta-analysis plan

We will undertake a meta-analysis of studies in which (i) there is a defined intervention or exposure, (ii) patients are receiving levothyroxine for hypothyroidism, (iii) there is an appropriate control group of levothyroxine treated patients that are not exposed to the intervention, and (iv) the primary outcome is determined by serum TSH. Studies will be included for meta-analysis if adequate information is provided, and meta-analysis of a topic will only proceed if there are sufficient numbers of relevant publications for the analysis to be meaningful. Studies will comprise randomised controlled trials as well as observational data with well-characterised control groups, but controlled trials and observational data will be pooled separately. Potential categories of interventions that will be assessed for meta-analysis include the optimal timing of levothyroxine administration (e.g. fasting vs non-fasted or morning vs bed time administration), the effect of concomitant drugs such as metformin and anti-epileptic medications on TSH levels, and the impact of co-morbidities and their treatment on the adequacy of levothyroxine therapy as determined by TSH levels.

We will determine the pooled difference in TSH expressed in mU/L (standardised mean difference with 95% confidence intervals) before and after the intervention. Unadjusted and adjusted effect sizes will be derived using a random effects model and inverse variance method. Heterogeneity across study results within each category will be assessed with the *I* ^*2*^ test for heterogeneity which will be graded as follows: *I* ^2^ values of 0%, no heterogeneity, 25–50% moderate heterogeneity and 50% high heterogeneity [26]. We will also assess publication bias with the Egger test which will be represented graphically using funnel plots of the standardised mean difference vs the standard error [27]. However, tests for bias will only be used if there are at least 10 studies in the meta-analysis, according to the Cochrane handbook for systematic reviews of interventions [28]. Statistical analysis will be undertaken with the Review Manager Software, version 5.2, The Nordic Cochrane Centre, The Cochrane Collaboration, 2012 [29].

## Discussion

This review seeks to further the understanding of the variety of factors that affect the adequacy of thyroid hormone replacement in patients diagnosed with hypothyroidism. The aim is to provide evidence to explain why patients experience TSH levels that are outside the normal reference range and to help healthcare professionals to better target areas to improve TSH control. Understanding the effects of various factors on TSH levels may help to comprehend why certain individuals are unhappy with their levothyroxine treatment.

This protocol has defined the search strategy to achieve the aims of the systematic review, how results will be evaluated, and how bias (Cochrane handbook [24]) and quality of evidence (GRADE) will be assessed. We have described patients, interventions, comparisons, outcomes, and study design (PICOS). We chose TSH levels as our primary outcome, and we acknowledge and will assess the effects upon T4 and T3 levels. Other significant secondary outcomes to be addressed include mortality, morbidity, quality of life, treatment complications, adverse effects, physical functioning and social functioning. Evidence from all potential studies will be initially accepted to give a broad approach that is necessary for our review.

The systematic review will be reported according to the PRISMA guidelines. This will be the first systematic review to focus on quantifying the effects of clinical, behavioural and pharmacogenomic factors on TSH levels in patients with primary hypothyroidism or subclinical hypothyroidism. Findings will be disseminated at conferences and in professional and peer-reviewed journals.

## Additional file

Additional file 1: Word document (62 KB). Preferred Reporting Items for Systematic Reviews and Meta-Analyses: The PRISMA statement. This file contains a 27-item checklist of recommended items to include in a systematic review and meta-analysis. (DOC 62 kb)

## Abbreviations

DIO2: Type 2 deiodinase
GRADE: Grading of Recommendations Assessment, Development, and Evaluation
NRS: Non-randomised studies
OR: Odds ratios
PICOS: Participants, interventions, comparators, outcomes, and study design
PRISMA: Preferred Reporting Items for Systematic Reviews and Meta-Analyses
QoL: Quality of life
RCTs: Randomised controlled trials
RR: Relative risks
SCH: Subclinical hypothyroidism
T3: Triiodothyronine
T4: Thyroxine
TSH: Thyroid stimulating hormone
